# Part-time Versus Full-time Spectacles for Myopia Control (ParMA Study): A Randomized Clinical Trial

**DOI:** 10.7759/cureus.25995

**Published:** 2022-06-16

**Authors:** Efthymia Prousali, Anna-Bettina Haidich, Anna Dastiridou, Argyrios Tzamalis, Nikolaos Ziakas, Asimina Mataftsi

**Affiliations:** 1 2nd Department of Ophthalmology, School of Medicine, Faculty of Health Sciences, Aristotle University of Thessaloniki, Thessaloniki, GRC; 2 Department of Hygiene, Social-Preventive Medicine and Medical Statistics, School of Medicine, Faculty of Health Sciences, Aristotle University of Thessaloniki, Thessaloniki, GRC

**Keywords:** childhood, refractive errors, part-time correction, spectacles, myopia control, myopia

## Abstract

Introduction

To compare myopia progression in school-aged children of Caucasian origin wearing part-time vs. full-time full correction with single-vision spectacles.

Methods

This prospective, randomized controlled trial included 30 children with bilateral myopia, who received either full-time or part-time treatment with single-vision spectacle lenses. Myopia progression was assessed as the mean change in cycloplegic spherical equivalent refraction (SE), mean change in axial length (AL), and mean change in sub-foveal choroidal thickness (SChT), over a 12-month follow-up period.

Results

A total of 32 eyes were treated with part-time single-vision spectacles (intervention group) and 28 eyes with full-time single-vision spectacles (control group), respectively. The part-time treated group reported no spectacle use during near-work activities for a mean of 6.2 hours/day. At the 12-month assessment, there was no significant difference between part-time and full-time correction groups in mean SE change (MD: -0.05 D, 95% CI: -0.50 - 0.39 D; *P* 0.81), in mean AL change (MD: -0.07 mm; 95% CI: -0.20 - 0.06 mm; P 0.30), and in mean SChT change (MD: -11.45 μm; 95% CI -22.60 - 14.16 μm; P 0.67).

Conclusion

Myopia progression in Caucasian children treated with part-time, single-vision spectacle use was not different compared to full-time, single-vision spectacle use, over a 12-month follow-up period.

## Introduction

Myopia is a visual disorder that has reached epidemic proportions globally, representing an emerging public health concern. According to WHO, myopia is defined by an objective refractive error greater than -0.50 D in either eye [1]. Myopia typically begins to develop from about six years of age and exhibits faster rates of progression below 10 years of age [2]. Myopia control at an early stage is of great clinical significance, as shortsightedness has been associated with secondary blinding conditions, including myopic maculopathy, choroidal neovascularization, glaucoma, cataract, and retinal detachment [3].

Investigation of myopia etiopathogenesis has been continuous during the past years, though much is yet unknown. Research in animal models supports the concept that myopia development may be precipitated by alterations in accommodative function. Emmetropization appears to be disrupted by hyperopic retinal blur occurring in the presence of near stimuli. This blur provokes an increased demand for accommodation, in order to bring the near image into optimal focus. Retinal defocus as a result of underaccommodation is believed to eventually drive ocular growth and myopia development [4,5].

So far, treatments that have demonstrated efficacy in myopia control include atropine eyedrops and optical interventions such as orthokeratology and multifocal lenses with novel designs. Notably, the financial burden of myopia is marked, as annual costs for its treatment are estimated to be greater than for other ocular and non-ocular chronic conditions. Also, visual impairment resulting from myopia or its complications appears to significantly affect the quality of life of myopic individuals.

Spectacles remain a non-invasive and accessible treatment option that has been routinely prescribed for the correction of refractive errors on a worldwide scale. A number of existing studies have explored the impact of under-correction on myopia progression, in an attempt to cause myopic defocus and eliminate the accommodative demand in near activities. Nonetheless, contradictory findings have been reported, from restriction up to worsening of myopia progression in response to under-correction [6-15]. In addition, evidence regarding the impact of part-time spectacle use on myopia progression is scarce. In view of this, we conducted this study to investigate the impact of part-time compared to full-time myopia correction on a pediatric population of Caucasian origin.

## Materials and methods

Study design

A prospective, randomized controlled trial (RCT) on school-aged myopic children was conducted from September 2019 to June 2021. Participants were randomized to receive either full-time or part-time full myopia treatment with single-vision spectacle lenses in both eyes, at an allocation ratio of 1:1, for one year. Informed written consent was obtained from the child’s parent or guardian as well as verbal assent from each child prior to enrolment. This study adhered to the tenets of the Declaration of Helsinki and was approved by the institutional review board of Papageorgiou General Hospital, Thessaloniki, Greece (Δ3β/32191). This trial is registered in the National Institutes of Health U.S. National Library of Medicine/clinicaltrials.gov (NCT04854447). Ethical approval was obtained from the Committee for Bioethics and Ethics, Aristotle University of Thessaloniki, Faculty of Health Sciences (2.68/27.2.2019).

Eligibility criteria

We prospectively recruited children with myopia who attended the pediatric ophthalmology outpatient clinic of the 2nd Department of Ophthalmology, Papageorgiou General Hospital, Thessaloniki, Greece. All participants met the following eligibility criteria: children aged four to 16 years old with best-corrected visual acuity of 0.1 logMAR or better in each eye, cycloplegic spherical equivalent (SE) refractive error range of -0.50 D to -6.00 D in each eye, astigmatism of -1.50 D or less in each eye, and anisometropia of 1.50 D or less. Exclusion criteria were the presence of ocular disorders, including strabismus, amblyopia, cataract, or lens dislocation; systemic disorders that could influence visual function and development, including Down syndrome and Marfan syndrome; history of premature birth at a gestational age of less than 37 weeks; and allergy to cyclopentolate.

Study procedures

Children were allocated with concealment into the part-time or the full-time spectacle treated group, according to a random assignment produced by a computer-generated randomization list. Owing to the nature of the assigned treatments (full-correction single-vision spectacles with part-time or full-time wear), neither participants nor investigators were blinded. Participants who were randomized into the part-time treated group were instructed to abstain from spectacle use during near-work activities and were provided with a detailed calendar to mark the daily hours they remained without correction. Instructions given in the intervention group were intended to prevent increased accommodative requirements, while aligning as possible with the real-life setting of participants, allowing them to use their glasses for optimal distance vision.

Best-corrected visual acuity (BCVA) was assessed on the logMAR scale using the Early Treatment Diabetic Retinopathy Study (ETDRS) chart at 4 m in a retro-illuminated box. Refraction was assessed by an autorefractor (Nidek Co. Ltd, Tokyo, Japan), prior to and post-cycloplegia, five times consecutively, and the average was analyzed. Cycloplegia was performed by instillation of 1% cyclopentolate drops twice, at a five-minute interval, and measurements were obtained 40 minutes after the second instillation. Axial length (AL) measurements were obtained with IOL Master® 500 (Carl Zeiss Meditec, Jena, Germany), with five readings averaged, and sub-foveal choroidal thickness (SChT) was assessed with spectral-domain optical coherence tomography (SD-OCT) prior to cycloplegia using Optovue (Optovue Inc, Freemont CA, USA). Keratometry readings were obtained for each eye with Nidek Co. Ltd, Tokyo, Japan. Accommodative amplitude was evaluated binocularly using a Royal Air Force (RAF) Rule. Time spent near work, physical activity, and outdoor exposure of participating children were also recorded. During each visit, a thorough discussion took place where patients and patient guardians were given the opportunity to report any health issues, or potential adverse events.

Outcome measures

The primary outcome is myopia progression. This was assessed as the mean change in cycloplegic SE and as the mean AL change, observed over six months and over one year. The secondary outcome included mean SChT change over six months and over one year.

Statistical analysis

IBM Corp. Released 2019. IBM SPSS Statistics for Windows, Version 26.0. Armonk, NY: IBM Corp was used for our analyses. Categorical variables were presented with frequencies and percentages and compared using the chi-square test. Continuous variables were presented with mean and standard deviation or median and interquartile ranges (Q1, Q3) depending on ascertainment of the normality, while for comparisons between the treatment groups an independent samples t-test or Mann-Whitney U test was used. To investigate potential risk factors associated with myopia progression, linear regression analysis was performed. In order to include data from both eyes of enrolled patients and due to our small sample size, we performed an analysis using the mean of the two values [16]. A two-sided p-value less than .05 was considered statistically significant. All analyses were performed on an intention-to-treat basis and multiple imputations were used for missing values. The sample size was conservatively estimated, assuming a mean difference of 0.35 D, with a standard deviation (SD) of 0.30 D between the intervention and control groups. Based on this effect size and considering an attrition rate of 15%, 15 participants in each group would be needed, given a power of 80% and a significance level of 5% [17].

## Results

**Figure 1:**
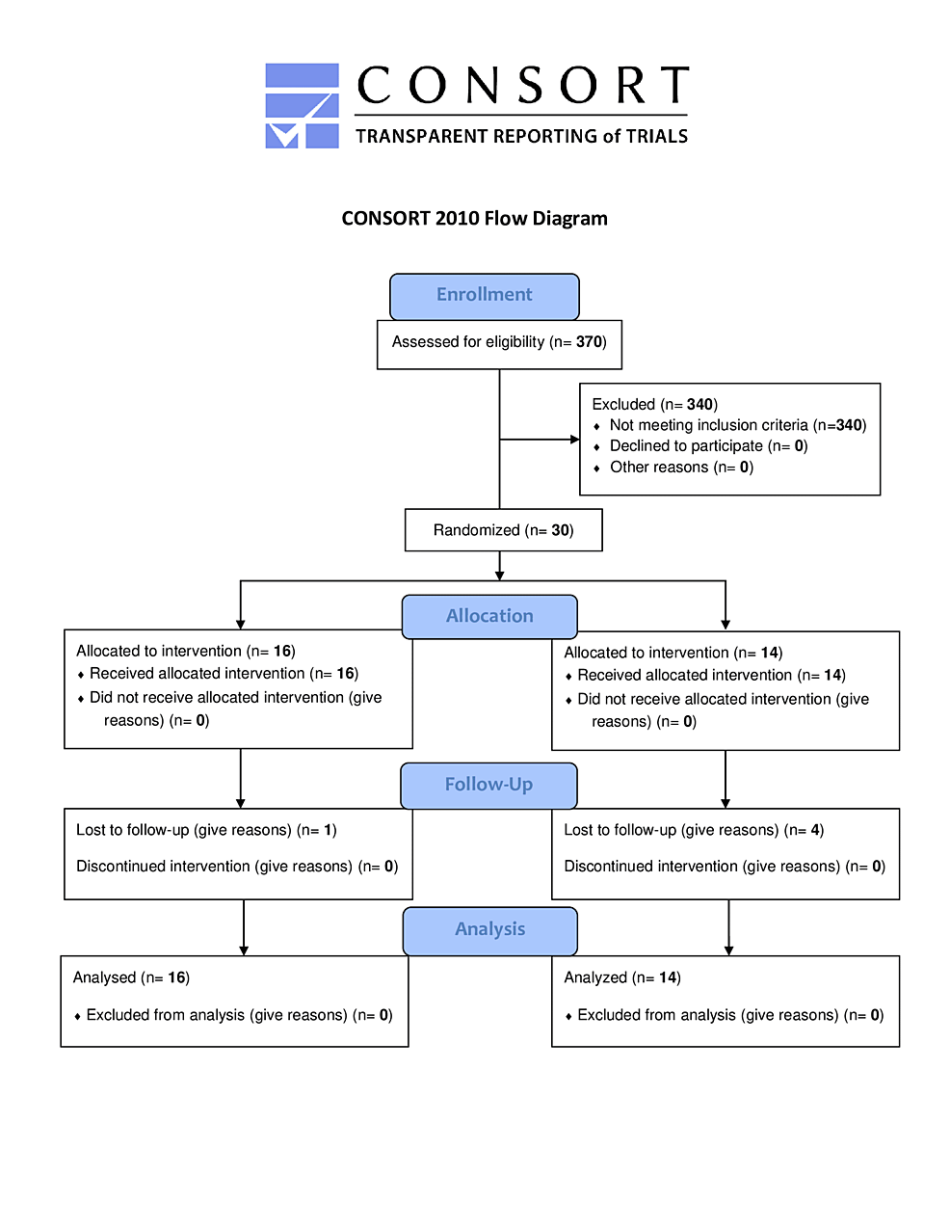
CONSORT Flow Diagram

A total of 30 children, 16 in the part-time (intervention group) and 14 in the full-time single-vision spectacle treatment group (control group), respectively, met the eligibility criteria and were recruited into this study (Figure 1). At the initial visit, there was no difference between the groups in terms of age, BCVA, weight, height, keratometry readings, and accommodative amplitude (Table 1). Overall, boys accounted for 18/30 participants (60%) and the mean age was 11.6 (2.2) years. The part-time treated group reported no spectacle use for a mean of 6.2 (3.5) hours/day (Table 2). Although refractive error and female gender were significantly higher in the full-time correction group than in the part-time correction group at recruitment [cycloplegic SE; mean difference (MD): 1.18, 95% confidence interval (CI):0.18 - 2.20; P 0.029], baseline AL and SChT were not different between the two groups (Table 3).

**Table 1:** Baseline characteristics of participants BCVA-best-corrected visual acuity; K1-flattest keratometry value; K2-steepest keratometry value; mean K-mean keratometry value; logMAR-logarithm of the minimum angle of resolution; N/A-not applicable; SD-standard deviation; Q1-first quartile; Q3-third quartile. Independent t-test; *Chi-square test; **Mann-Whitney U test.

Characteristics	Part-time correction group (n=16)	Full-time correction group (n=14)	p-value
Gender*			0.01
Male (%)	14 (87.5%)	4 (28.6%)	
Female (%)	2 (12.5%)	10 (71.4%)	
Mean age (SD) at initial examination in years	11.6 (2.5)	11.5 (2.0)	0.88
Mean weight (SD) in kg	56.1 (19.0)	49.4 (14.7)	0.39
Mean height (SD) in cm	155.3 (16.1)	151.7 (8.4)	0.56
Mean age at myopia onset (SD) in years	9.2 (2.4)	8.1 (2.1)	0.22
Mean gestational age (SD) in weeks	39.1 (1.6)	38.1 (1.9)	0.92
Median birth weight (Q1, Q3) in grams**	3460.0 (3188, 3740)	3295.0 (2800, 3606)	0.21
Paternal smoking*			0.19
Yes (%)	4 (25%)	8 (57%)	
No (%)	12 (75%)	6 (43%)	
Maternal smoking*			0.52
Yes (%)	3 (19%)	2 (14%)	
No (%)	13 (81%)	12 (86%)	
Maternal smoking at pregnancy*			0.18
Yes (%)	3 (19%)	1 (7%)	
No (%)	13 (81%)	13 (93%)	
Parental myopia*			0.74
None	7 (43.5%)	5 (36%)	
One	7 (43.5%)	8 (57%)	
Both	2 (13%)	1 (7%)	
Dominant eye*			0.63
Right	10 (63%)	9 (64%)	
Left	5 (31%)	5 (36%)	
Indefinite	1 (1%)	0	
Mean reading distance (SD) in cm	33.7 (4.9)	32.9 (8.1)	0.73
Median distance BCVA (Q1, Q3) in logMAR**	0.0 (0.0, 0.01)	0.0 (0.0, 0.03)	0.97
Median near BCVA (Q1, Q3) in logMAR**	0.0 (0.0, 0.1)	0.0 (0.0, 0.01)	0.11
Mean K1 (SD) in diopters	43.0 (0.20)	43.2 (0.17)	0.79
Mean K2 (SD) in diopters	44.0 (0.19)	44.2 (0.15)	0.72
Mean K (SD) in diopters	43.5 (0.19)	43.7 (0.16)	0.77
Median accommodative amplitude (Q1, Q3) at initial examination in diopters**	9.1 (8.3, 11.1)	9.1 (8.9, 12.5)	0.61

**Table 2:** Time participants spent on daily activities during follow-up SD-standard deviation; Q1-first quartile; Q3-third quartile. Independent t test; * Mann-Whitney U test.

Activities	Part-time correction group (n=16)	Full-time correction group (n=14)	p-value
Mean time/day spent on near work activities (SD) in hours	6.2 (3.5)	5.4 (2.8)	0.25
Median outdoors exposure/day (Q1, Q3) in hours*	0.8 (0.5, 1.5)	0.75 (0.5, 1.25)	0.35
Median time spent on sport activities/day (Q1, Q3) in hours*	0.5 (0.5, 1.0)	0.5 (0.0, 1.0)	0.45

**Table 3:** Participant baseline myopia, axial length, and sub-foveal choroidal thickness AL-axial length; CI-confidence interval; SChT-sub-foveal choroidal thickness; SD-standard deviation; SE-spherical equivalent. Independent t-test.

Variables	Part-time correction group (n=16)	Full-time correction group (n=14)	Mean difference	95% CI	p-value
Mean cycloplegic SE (SD) in diopters	2.3 (1.11)	3.52 (1.51)	1.18	0.18 - 2.20	0.029
Mean AL (SD) in mm	24.39 (1.1)	24.51 (0.9)	0.11	-0.64-0.86	0.76
Mean SChT (SD) in μm	232.4 (27.7)	231.8 (26.3)	0.52	-19.7 - 20.7	0.96

Twenty-five children (83.3%) completed the one-year follow-up. Five children failed to attend the 12-month follow-up, 1 (6.3%) in the part-time and four (28.6%) in the full-time treatment group, owing to geographic distance and restrictions imposed due to COVID -19 pandemic. At the one-year assessment, there was no difference between part-time and full-time correction groups in mean SE change (MD: -0.05 D, 95% CI: -0.50 - 0.39 D; P 0.81). Accordingly, mean AL change did not differ for the intervention group and control group (MD: -0.07 mm; 95% CI: -0.20 - 0.06 mm; P 0.30). Mean SChT change was similar between part-time and full-time treatment group (MD: -11.45 μm; 95% CI -22.06 - 14.16 μm; P 0.67) (Table 4). The complete case analysis resulted in similar estimates in terms of statistical significance compared to the imputed analysis presented. None of the participants developed astigmatism greater than 1.5 D, in either eye, during the follow-up. No adverse events related to spectacle use were reported, in either group. Univariate linear regression analysis indicated that mean SE change was positively associated with time spent on near work activities per day (beta 1.3, P 0.015), and inversely related with gestational age (beta -18.3, P 0.009), in the part-time treated group (Tables 5, 6).

**Table 4:** Primary and secondary outcomes at follow-up visits AL-axial length; SChT-sub-foveal choroidal thickness; SE-spherical equivalent. Independent t-test.

Variables	Follow-up period	Part-time correction group (n=16)	Full-time correction group (n=14)	Mean Difference	95% CI	p-value
Mean cycloplegic SE change (Std. Error) in diopters	6 months	-0.58 (0.10)	-0.45 (0.16)	0.13	-0.29 - 0.55	0.54
Mean cycloplegic SE change (Std. Error) in diopters	12 months	-0.67 (0.13)	-0.73 (0.19)	-0.05	-0.50 - 0.39	0.81
Mean AL change (Std. Error) in mm	6 months	0.16 (0.04)	0.15 (0.03)	0.01	-0.10 - 0.12	0.84
Mean AL change (Std. Error) in mm	12 months	0.25 (0.05)	0.32 (0.05)	-0.07	-0.20 - 0.06	0.30
Mean SChT change (Std. Error) in μm	6 months	-6.84 (9.72)	8.85 (11.53)	-15.69	-47.25 - 15.87	0.32
Mean SChT change (Std. Error) in μm	12 months	-11.45 (5.12)	-7.50 (7.74)	-3.95	-22.06 - 14.16	0.67

**Table 5:** Regression analysis of risk factors for the part-time correction group (n=16) AA-accommodative amplitude; AL-axial length; BCVA-best-corrected visual acuity; CI-confidence interval; logMAR-logarithm of the minimum angle of resolution; SChT-sub-foveal choroidal thickness; SE-spherical equivalent.

Variable	beta (95% CI)	p-value
Gender (Male/Female)	11.0 (-52.4, 74.5)	0.73
Age at baseline	2.4 (-8.0, 12.8)	0.65
Weight at baseline	0.06 (-1.7, 1.8)	0.95
Height at baseline	0.4 (-1.3, 2.1)	0.64
Age at myopia onset	1.7 (-9.0, 12.5)	0.75
Gestational age	-18.3 (-32.0, -4.6)	0.009
Birth weight	-0.01 (-0.08, 0.07)	0.87
Paternal smoking	-13.1 (-70.2, 44.1)	0.65
Maternal smoking	-9.5 (-73.0, 54.0)	0.77
Maternal smoking at pregnancy	-6.7 (-75.8, 62.4)	0.85
Presence of parental myopia	14.2 (-21.5, 49.9)	0.44
Time/day spent on near work activities	1.3 (0.2, 2.3)	0.015
Outdoor exposure/day	-0.8 (-3.5, 1.9)	0.55
Time spent on sport activities/day	1.6 (-4.1, 7.3)	0.56
Reading distance	1.0 (-4.9, 6.8)	0.74
Distance BCVA at baseline	-282.3 (-900.9, 336.4)	0.37
Near BCVA at baseline	-404.0 (-964.5, 156.5)	0.16
AA at baseline	1.6 (-9.2, 12.5)	0.77
Cycloplegic SE at baseline	-0.04 (-0.3, 0.2)	0.71
AL at baseline	-0.03 (-0.26, 0.2)	0.81
SChT at baseline	-0.22 (-1.16, 0.71)	0.64

**Table 6:** Regression analysis of risk factors for the full-time correction (control) group (n=14) AA-accommodative amplitude; AL-axial length; BCVA-best-corrected visual acuity; CI-confidence interval; logMAR-logarithm of the minimum angle of resolution; SChT-sub-foveal choroidal thickness; SE-spherical equivalent.

Variable	beta (95% CI)	p-value
Gender (Male/Female)	-31.4 (-108.2, 45.5)	0.41
Age at baseline	-2.4 (-18.8, 14.0)	0.77
Weight at baseline	-1.1 (-3.3, 1.0)	0.31
Height at baseline	-0.3 (-4.2, 3.5)	0.86
Age at myopia onset	3.9 (-12.0, 19.8)	0.63
Gestational age	14.8 (-5.1, 34.6)	0.15
Birth weight	-0.03 (-0.12, 0.06)	0.52
Paternal smoking	35.9 (-31.8, 103.6)	0.29
Maternal smoking	10.9 (-81.0, 102.7)	0.81
Maternal smoking at pregnancy	-42.2 (-194.3, 109.8)	0.57
Presence of parental myopia	-17.0 (-94.6, 60.6)	0.65
Time/day spent on near work activities	0.50 (-1.1, 2.1)	0.54
Outdoor exposure/day	-4.5 (-10.0, 1.0)	0.11
Time spent on sport activities/day	0.15 (-0.65, 0.95)	0.71
Reading distance	-0.04 (-5.4, 5.3)	0.99
Distance BCVA at baseline	324.1 (-826.8, 1475.0)	0.58
Near BCVA at baseline	436.2 (-469.6, 1342.1)	0.34
AA at baseline	-3.7 (-21.2, 13.9)	0.67
Cycloplegic SE at baseline	0.02 (-0.21, 0.24)	0.90
AL at baseline	0.24 (-0.16, 0.60)	0.18
SChT at baseline	0.46 (-0.8, 1.7)	0.47

## Discussion

In this randomized clinical trial comparing part-time and full-time wear of single-vision spectacles in school-aged children of Caucasian origin, myopia progression was not found to be different in the two groups, over a 12-month follow-up period. Axial elongation and choroidal thinning were also similar in the two groups during the same period. Myopia progression within each group in the 12-month follow-up was also estimated. In particular, the intervention group demonstrated a mean change of 0.67 D in refraction and 0.25 mm in axial length, while the control group showed a mean change of 0.73 D in refraction and 0.32 mm in axial length. These outcomes corroborate the findings reported by Tideman et al., who observed a mean annual change of 0.34 mm in axial length, with a range of 0.17 to 0.53 mm, in a cohort of 279 myopic children of European origin [18].

To date, three studies exploring part-time single-vision spectacle use in myopia progression have been published several years ago, reporting conflicting findings [15,19,20]. Parsinnen et al. in their RCT compared three groups of overall 240 myopic schoolchildren, including full-time correction, full-time correction worn only for distance vision, and bifocal lenses. The authors found no difference in the right eye, and significant myopia progression in the left eye in the distance-corrected compared to the fully corrected group, in a follow-up period varying between two and 5.1 years. In the same study, compliance was categorized as full or partial, depending on the child’s perception. Interestingly, in this study, a partly or non-compliant full-time corrected patient could supposedly be regarded as a distance-corrected patient, which may not have been clearly accounted for in the study design [20]. In their longitudinal, non-randomized study, Ong et al. found no difference in myopia progression between distance-corrected and full-time corrected myopic children, after assessment of four treatment groups in a sample of overall 43 children [15]. This finding is consistent with the results of our study, although the small sample size in each group is acknowledged as a limitation by Ong et al. An earlier study by Tokoro and Kabe reported greater myopia progression in the full-time compared to the part-time spectacle-treated group; however small sample size and possible co-existing treatment modalities applied to some participants on top of spectacle use, have rendered their findings equivocal [19].

It has been well-established that emmetropization is regulated by visual signals, which translate into a corresponding response in ocular growth. When full-time myopia correction is used, an inadequate accommodative response for a near target would extend the presence of hyperopic defocus, thereby triggering axial elongation and myopia progression. In keeping with this presumption, full-time corrected participants would be expected to demonstrate greater myopia progression compared to the part-time corrected group, given that myopes may exhibit an accommodative lag at near distances [21]. Nonetheless, this was not supported by our findings. No significant difference was observed between the two study groups in refraction or in axial elongation, and this finding is in agreement with outcomes reported by Ong et al. and Parsinnen et al. for the right eye [15,20]. Axial elongation resulting from stimulation of accommodation has been associated with extension and thinning of the choroid, which also reflects myopia progression and is increasingly described by myopia control studies [22]. In line with our other findings, we detected a similar reduction in sub-foveal choroidal thickness in both cases and controls, which substantiated that myopia progression was not different between the two groups.

Myopia progression has been associated with a number of confounding conditions. The amount of near work has been reported to play a critical role in myopia development [23]. We found a significant positive correlation between time spent on near activities and myopia progression only in the part-time treated group (P 0.015). It is true that this group happened to have less myopia, as estimated by cycloplegic refractive measurements, than the group assigned to full-time spectacle wear, despite the randomization we undertook. Thus, in a way, these children were more at ease when not wearing their spectacles. One would have expected that the time spent on near work would contribute to a lesser extent to myopia progression in the intervention group, which was not corrected for near vision. We failed to demonstrate such a difference between the two groups, while the mean reading distance was similar in both cases and controls (P 0.73). Although the proportion of females and the level of myopia were higher in the control group owing to the small sample size, myopia progression was found similar between male and female participants in our sample (SE: P 0.61, AL: P 0.66, ChT: P 0.77), and gender was not correlated with the rate of myopia progression in either treatment group. Interestingly, data derived from the correction of myopia evaluation trial (COMET) illustrated that ocular growth is greater under the age of 10 in a cohort of 431 myopic children, and evidenced a similar amount of myopia and age of axial length stabilization in both sexes [24]. Since our sample had a mean age of 11.6 years, our data is apparently ahead of the curve of presumed fast myopia progression. On top of this, choroidal thickness has been reported to either remain unaffected or increase in response to pubertal maturation which may be more advanced in females [22]. Our observations would support the concept that myopia development demonstrates a multifactorial nature, which is influenced by a multiplicity of environmental factors in addition to suspected a genetic component.

In our study outdoors exposure was negatively associated with myopia progression in both groups, though it did not reach the level of statistical significance. This relationship has been broadly discussed by existing literature, with evidence concluding that increased outdoor activities appear to be related to reduced myopia development or progression [25,26]. Notably, we did not find a concurrent significant association between physical exercise and myopia progression. As illustrated by Thykjaer et al., an independent correlation between physical activity and myopia progression has not been proven. A number of studies have shown a reverse relationship between physical activity and myopia progression; however, a possible parallel effect of outdoor exposure should be taken into consideration [27]. Interestingly, gestational age was inversely associated with myopia progression in our part-time treated group (P 0.016). As reported by previous studies, term-born children are found to be hypermetropic, while premature children demonstrate myopia which shows a decreasing trend with increasing gestational age [28,29]. However, supportive evidence has not been consistently shown by other studies regarding refractive error development in school-aged children. One cross-sectional study that looked at the relationship between birth factors and refractive development reported that hypermetropia was associated with small gestational age Caucasian children [30]. Nonetheless, our participants were appropriate for gestational age; thus we could not reasonably compare these findings with our results. Given our relatively small number of participants, further investigation will be needed to explore the validity of our observed outcomes. In this study, no significant correlation was found for other parameters, including baseline refraction and axial length, age, birth weight, and parental myopia with the rate of myopia progression.

A key strength of our study is the prospective, randomized controlled trial design. This was combined with the use of cycloplegic refractive measurements for assessing the actual level of myopia, which is especially important when examining pediatric populations. Another strength compared to previous studies is that we also assessed choroidal thickness variation with myopia progression, which has been reported as an additional clinical biomarker of myopia development. A weakness was the sample size that confined us in terms of performing multiple regression analyses. Therefore, caution should be applied to the interpretation of our findings. Also, in our study as in any study assessing spectacle use, treatment compliance could only be evaluated based on patients’ and patient guardians’ reliability. For this reason, we attempted to minimize the subjectivity of reporting by using a calendar to mark time spent on a number of daily activities.

## Conclusions

It is widely recognized that the growing epidemic of myopia poses significant challenges in terms of trying to halt its occurrence and its rate of progression. Proper monofocal myopia correction remains clinically relevant. To our knowledge, this is the first RCT investigating part-time single-vision full myopia correction not worn near distances, using a calendar for recording the hours per day of spectacle use as accurately as possible and assessing parameters such as time spent near work, physical exercise, and outdoor exposure. Although our findings demonstrate that there is no clinical difference between part-time and full-time single vision spectacle use in the rate of myopia progression, further evidence on the effect of lenses on myopia development in pediatric populations is needed.
